# Pharmacy deserts and health inequities in St. Louis City: a geospatial analysis of access disparities and determinants

**DOI:** 10.1186/s12889-025-25910-3

**Published:** 2025-12-12

**Authors:** Antoine Brantley, Dominic Mosha, Bobie Williams, Quinn J. Hill, Shontae Fluelen, Marcus Howard, Elvin Geng, Matifadza Hlatshwayo-Davis

**Affiliations:** 1 City of St. Louis Department of Health, 1520 Market St., Room 4051, St. Louis, 63103 MO USA; 2 GreaterHealth Pharmacy & Wellness, St. Louis, MO USA; 3 Division of Infectious Diseases, Washington University School of Medicine, St. Louis, MO USA

**Keywords:** Pharmacy desert, Health equity, Healthcare access, Medication access, Health disparities

## Abstract

**Background:**

Pharmacy deserts, or areas lacking convenient access to pharmacies, can limit access to medications and reduce adherence. In U.S. cities, pharmacy deserts are disproportionately concentrated in racial/ethnic minority and low-income neighborhoods. This study examined the demographics and spatial distribution of pharmacy deserts in St. Louis City, Missouri, and their associations with sociodemographic and healthcare access determinants.

**Methods:**

Pharmacy deserts were defined as census tract block groups whose centroids were more than 1 mile from a pharmacy, or more than a half mile and located within a tract where at least 20% of households lacked vehicle access. Demographic and socioeconomic data came from the 2020 Decennial Census and the 2019–2023 American Community Survey. We assessed associations between block-group characteristics and pharmacy-desert status using chi-square tests; estimated prevalence ratios (PRs) with modified Poisson regression (log link) using robust standard errors clustered by census tract; and tested a race × income interaction with a cluster-robust Wald F test.

**Results:**

Among St. Louis City residents, 17.2% lived in pharmacy deserts. Black residents accounted for 80% of the pharmacy desert population, compared to 43% citywide. Pharmacy deserts were 9.53 times more prevalent in majority-Black block groups (prevalence ratio: 9.53; 95% CI: 3.17–28.6). Income significantly modified the race–desert association (cluster-robust Wald F(1, 103) = 4.53, *p* = 0.036; Table 3; Fig. 2). Within low-income census tracts, majority-Black block groups had over three times the prevalence of pharmacy deserts (PR = 3.23; 95% CI, 1.03–10.16; *p* = 0.045), whereas within high-income census tracts the disparity was larger (PR = 43.00; 95% CI, 5.08–364.21; *p* = 0.001) but imprecise given the small number of pharmacy deserts in higher-income areas. Lorenz curves showed that 90% of the pharmacy desert population was concentrated in just 28% of census tracts, most with Black populations exceeding 89%.

**Conclusion:**

Racial and socioeconomic spatial inequities are strongly associated with pharmacy desert prevalence in St. Louis City. These findings are guiding coordinated efforts between the City of St. Louis Department of Health and a community pharmacy to improve access and reduce inequities.

## Background

A rise in pharmacy closures across the U.S. is leaving many communities with limited access to medications and pharmacy services, particularly in areas already struggling with considerable healthcare barriers. Between 2009 and 2015, one in six urban pharmacies (*n* = 9,564) closed, with closures occurring twice as often in areas with higher proportions of low-income, uninsured, and publicly insured residents [1]. Recently published data from 2010 to 2021 found that nearly one-third of U.S. pharmacies closed during this period, with Black and Latino neighborhoods remaining the most vulnerable to closures, even after accounting for neighborhood and market characteristics [2]. Between 2021 and 2024, major U.S. retail pharmacy chains announced an additional 2,820 closures, with one chain filing for bankruptcy [3].

As these closures increase, so does the number of people living in “pharmacy deserts”. In urban settings, a pharmacy desert is typically defined as an area where residents live more than a mile from a pharmacy or more than a half mile if they lack reliable transportation. Two studies on pharmacy deserts in U.S. cities, covering the periods 2007–2015 and 2015–2020, found significantly higher numbers of pharmacy deserts in Black and Latino neighborhoods compared to White or diverse neighborhoods in urban areas. Black neighborhoods had the fewest pharmacies overall [1, 4, 5]. Similarly, a study examining the period from 2017 to 2021 revealed that populations residing in pharmacy deserts across the U.S. were more likely to have lower educational attainment, lower incomes, reduced health insurance coverage, and a higher proportion of racial or ethnic minorities compared to those living in areas with better pharmacy access. Pharmacy deserts have also been shown to be more prevalent in Medically Underserved Areas, areas characterized by factors such as a shortage of primary care providers, high infant mortality rates, and/or large elderly populations [4, 6–12].

Limited pharmacy access has been associated with barriers to medication adherence, particularly for elderly, low-income, and historically marginalized populations [8, 10]. Living in pharmacy deserts is associated with poorer chronic disease management and higher travel costs, especially in Medically Underserved Areas [13]. A study examining the relationship between pharmacy accessibility, utilization, and cost-related underuse of prescription medications in predominantly Black and Hispanic communities with low socioeconomic status in Chicago also found significant disparities. Residents with limited access to pharmacies were more likely to report cost-related underuse of prescription medications compared to those with a pharmacy located within one mile of their home. Furthermore, the study highlighted that low-income and uninsured individuals often traveled longer distances to obtain medications and avoided nearby independently owned community pharmacies due to the higher costs of similar medications [8].

St. Louis City displays many characteristics commonly associated with pharmacy deserts. Systemic policies, such as discriminatory real estate and mortgage lending practices, have historically concentrated Black residents in underinvested neighborhoods, leading to persistent socioeconomic disparities and limited access to healthcare resources [14, 15]. As of 2022, over a quarter of Black St. Louis residents lived below the federal poverty line, compared to 12% of White residents. Additionally, 13% of Black residents lived in areas of concentrated poverty, compared to just 2.1% of White residents [16, 17].

A 2022 geospatial analysis found stark disparities in pharmacy access across St. Louis City. Using 2020 Census data and pharmacy location mapping, the study found that nearly one-sixth of St. Louis residents lived more than a mile from a pharmacy, with Black residents disproportionately represented in pharmacy desert areas. In hot-spot areas where the average distance to the nearest pharmacy was highest, more than 90% of residents were Black and fewer than 5% were White [18].

The present study expands upon the prior geospatial analysis of pharmacy deserts in St. Louis City by providing a demographic and socioeconomic profile of the populations affected and examining the association between pharmacy desert prevalence and neighborhood racial and income composition. In addition, we apply Lorenz curves and Gini coefficients, to measure the magnitude of geographic inequality in pharmacy desert distribution.

## Methods

### Study area

The study area was St. Louis City, Missouri, which had 282,772 residents in 2023. Of these, 49.9% were White, 43.1% Black, 3.8% Asian, 3.2% another race or combination of races, and 5.4% Latino (any race) [19]. Median household income was $56,245, and 19.6% lived below the federal poverty level [20–22]. For context, in 2023, the federal poverty level was defined as an annual income of $14,580 for a single-person household and $30,000 for a family of four [23]. Among adults aged ≥ 25 years, 38.4% had a bachelor’s degree or higher [24].

### Population data used in analysis

Census tract block group–level population counts for total population, race, age, and sex were obtained from the 2020 Decennial Census [25, 26]. Census tract–level estimates for poverty status, Medicaid coverage, vehicle availability, ambulatory difficulty, and educational attainment were sourced from the 2023 American Community Survey (ACS) 5-year estimates [21, 27–29]. Although ACS estimates are available at the block-group level, small-area estimates for ACS socioeconomic indicators become increasingly uncertain as the geographic units get smaller. Prior work has documented substantial margins of error and high coefficients of variation for ACS census tract- and especially block-group–level estimates [30–32]. Consistent with this guidance, we examined the ACS margins of error and coefficients of variation for our study area and found that tract-level estimates were reasonably precise, whereas block-group estimates for the same indicators often had unacceptably large margins of error. Because ACS block-group estimates for these socioeconomic indicators often had unacceptably large margins of error, we used tract-level ACS estimates and assigned each block group the values of its parent tract. This approach treats census tracts as the primary unit for measuring area-level socioeconomic context, consistent with recent comparative evidence that tract-level SES measures perform at least as well as block-group measures and better than ZIP- and county-level measures in predicting health outcomes, while still allowing us to retain the finer spatial resolution of block-group–defined pharmacy deserts [33].

### Pharmacy desert mapping

A shapefile of licensed Missouri pharmacies (June 2023) was obtained from the Missouri Spatial Data Information Service and updated in August 2024 to remove two recent closures in Northeast and Southwest St. Louis [34]. Pharmacies outside St. Louis were included if within one mile of the city limits. Although the city borders Illinois, a Google search confirmed that no Illinois pharmacies were located within one mile of the city limits. A second shapefile of city boundaries, tracts, and block groups was obtained from the U.S. Census Bureau [35–37] and merged with ACS data.

Pharmacy deserts were defined as block groups whose centroids were greater than one mile from a pharmacy or greater than a half mile and in a census tract where at least 20% of households lacked a vehicle. Straight-line (Euclidean) distances were calculated in ArcGIS Pro using the Spatial Join tool. A similar approach was used in another study mapping pharmacy deserts in St. Louis City [18]. On the map, pharmacy desert block groups were shaded alongside majority-Black block groups, where Black residents made up more than 50% of the population, and low-income census tracts, where 20% or more of residents lived below the poverty line or had a median family income below 80% of the metropolitan area’s median. Our definition of low-income census tracts follows the same criteria used by the U.S. Department of Agriculture for food desert designation, which identifies low-income census tracts with limited access to affordable food, and by the federal Opportunity Zone program, which designates economically distressed census tracts eligible for investment incentives [38, 39]. All geospatial analyses were conducted using ArcGIS Pro 3.2.1.

### Statistical analysis

Chi-square tests and descriptive statistics compared demographics and socioeconomic characteristics between pharmacy desert and non-desert areas (*p* < 0.05). We estimated prevalence ratios (PRs) using modified Poisson regression (log link) with robust standard errors clustered at the census-tract level. Primary predictors were block-group racial composition (majority-Black vs. non-majority-Black) and census-tract income (low vs. high). A similar approach was used in prior work to assess the prevalence of pharmacy deserts across large cities in the U.S. by neighborhood racial and income composition [1]. We then tested effect modification by income by adding a race × income interaction term and evaluating it with a cluster-robust Wald F test. When the interaction was present, we reported stratum-specific PRs from separate stratified models. Analyses were performed in RStudio 2024.09.0.

### Sensitivity analyses (alternative access metrics, density, and additional sociodemographics)

To assess the stability of our findings, we conducted sensitivity analyses that substituted an alternative access metric (pharmacies per capita), evaluated the effect of population density, and examined the impact of adding additional sociodemographic covariates to the regression models. Pharmacies per capita was calculated for each block group as the number of pharmacies within a 1-mile radius of the block-group centroid per 10,000 residents. Population density was defined as total population divided by land area (km²). For regression, both pharmacies-per-capita and density were log-transformed; density effects are reported per 1 standard deviation (SD) increase in log density. Univariate comparisons used Welch’s t-tests and Wilcoxon rank-sum tests for group differences in log-transformed measures, and χ² tests to compare quintile distributions (Q1–Q5). We then re-estimated modified Poisson models with robust standard errors clustered by census tract under two specifications: Model A added the alternative access metric (or density) to the core terms—block-group race, census-tract income, and their interaction—whereas Model B additionally included educational attainment, Medicaid coverage, ambulatory difficulty, age, and sex composition.

### Additional sensitivity analyses (distance bands and vehicle-access cutoffs)

We conducted two additional sensitivity analyses to evaluate the robustness of the pharmacy-desert definition. First, we varied the fixed distance band used to define deserts, classifying block groups as deserts if their centroids were more than 0.5, 0.75, 1.0, 1.25, 1.5, or 2.0 miles from the nearest pharmacy, without incorporating vehicle access. For each specification, we estimated overall prevalence and unadjusted prevalence ratios (PRs) with 95% CIs using modified Poisson regression (log link) with robust standard errors clustered by census tract, comparing majority-Black vs. non-majority-Black block groups and low- vs. high-income tracts (and, secondarily, race stratified within income groups). When a distance band yielded zero deserts in the reference category, PRs were not estimable; in those instances we reported descriptive prevalences and noted non-estimability due to zero cells. Second, we examined the household vehicle-access cutoff in the composite definition by repeating the main analysis with thresholds of 10%, 15%, 20% (primary), 25%, and 30% of households without a vehicle. For each threshold, we recalculated desert prevalence and estimated the same unadjusted PRs with 95% CIs.*Lorenz Curves*.

To assess geographic inequality, we first aggregated the block-group–level pharmacy desert indicator to the census-tract level. For each census tract, we calculated the number of residents living in pharmacy-desert block groups within that tract. We then constructed Lorenz curves at the census-tract level by plotting the cumulative share of all residents living in pharmacy deserts (Y-axis) against census tracts ranked from lowest to highest number of residents living in pharmacy-desert block groups (X-axis). Each census tract was weighted according to its total population size. Curves that bow further from the 45-degree line indicate greater concentration of pharmacy deserts in fewer census tracts. Gini coefficients were calculated to quantify this inequality, with values closer to 1 reflecting higher concentration. Lorenz curves have been used to investigate geographic inequities in various studies, including a recent study that analyzed racial disparities in COVID-19 testing and hospitalizations [40]. Lorenz curves and Gini coefficients were generated in RStudio version 2024.09.0.

## Results

### Demographic and socioeconomic profile

As shown in Table 1,7.2% (51,781 residents) of the population in St. Louis City resided in pharmacy deserts. Pharmacy desert population characteristics varied significantly (*p* < 0.001) across demographic and socioeconomic groups, with notable differences in racial composition, transportation access, income, Medicaid enrollment, and education levels. Black residents comprised 80.0% of the population living in pharmacy deserts, despite making up only 43.0% of the general city population. The proportion of individuals in carless households was higher in pharmacy deserts (30.6%) compared to the general population (18.5%). Individuals with annual incomes below the federal poverty level made up 23.5% of the pharmacy desert population, versus 13.9% of the general population. Medicaid recipients constituted 39.4% of the pharmacy desert population compared to 23.3% of the general population. Additionally, individuals without a Bachelor’s degree accounted for 81.2% of the pharmacy desert population, compared to 60.7% of the general population.

**Table 1:** Population demographic and socioeconomic characteristics of block groups by pharmacy desert status in St. Louis City [21, 23–29, 34]

Characteristic	Total population	Pharmacy desert^a^	*p*-value
Total	301,578 (100%)	51,781 (100%)	N/A
Race^b^			
Black or African alone	129,814 (43.0%)	41,434 (80.0%)	< 0.001
Non-Black	171,764 (57.0%)	10,347 (20.0%)	
Age^b^			
Below 65	261,030 (86.6%)	43,909 (84.8%)	< 0.001
65 and over	40,548 (13.4%)	7,872 (15.2%)	
Sex^b^			
Female	154,238 (51.1%)	27,258 (52.6%)	< 0.001
Male	147,340 (48.9%)	24,523 (47.4%)	
Household vehicle availability^c^			
Has one or more vehicles	81.5%	69.4%	< 0.001
Does not have a vehicle	18.5%	30.6%	
Annual income below FPL^c, d^			
Yes	13.9%	23.5%	< 0.001
No	86.1%	76.5%	
Medicaid coverage^c^			
Has coverage	23.3%	39.4%	< 0.001
Does not have coverage	76.7%	60.6%	
Ambulatory difficulty status^c^			
Has ambulatory difficulty	10.9%	17.0%	< 0.001
Does not have an ambulatory difficulty	89.1%	83.0%	
Educational attainment^c, e^			
No Bachelor’s degree	60.7%	81.2%	< 0.001
Bachelor’s degree or higher	39.3%	18.8%	

Abbreviations: *No.* number, *pop.* population, *FPL* Federal poverty level

^a^ Pharmacy deserts were defined as block-group centroids ≥ 1 mile from the nearest licensed pharmacy or ≥ 0.5 mile where ≥ 20% of households lack a vehicle [24, 34]

^b^ The demographic and socioeconomic variables for race and ethnicity, sex, and age group were drawn from the 2020 Decennial Census [25, 26]

^c^ Characteristics such as household vehicle availability, income below the federal poverty level, and Medicaid coverage were based on the 2023 American Community Survey (ACS) 5-year estimates. To maintain data integrity, percentages were not applied to 2020 Decennial Census totals given differences in methodology and reference periods [21, 27–29]

^d^ In 2022, the federal poverty level was $13,590 annually for a household of 1 and $27,750 for a household of 4 [23]

^e^ For population aged 25 and older

### Prevalence ratios

**Fig. 1:**
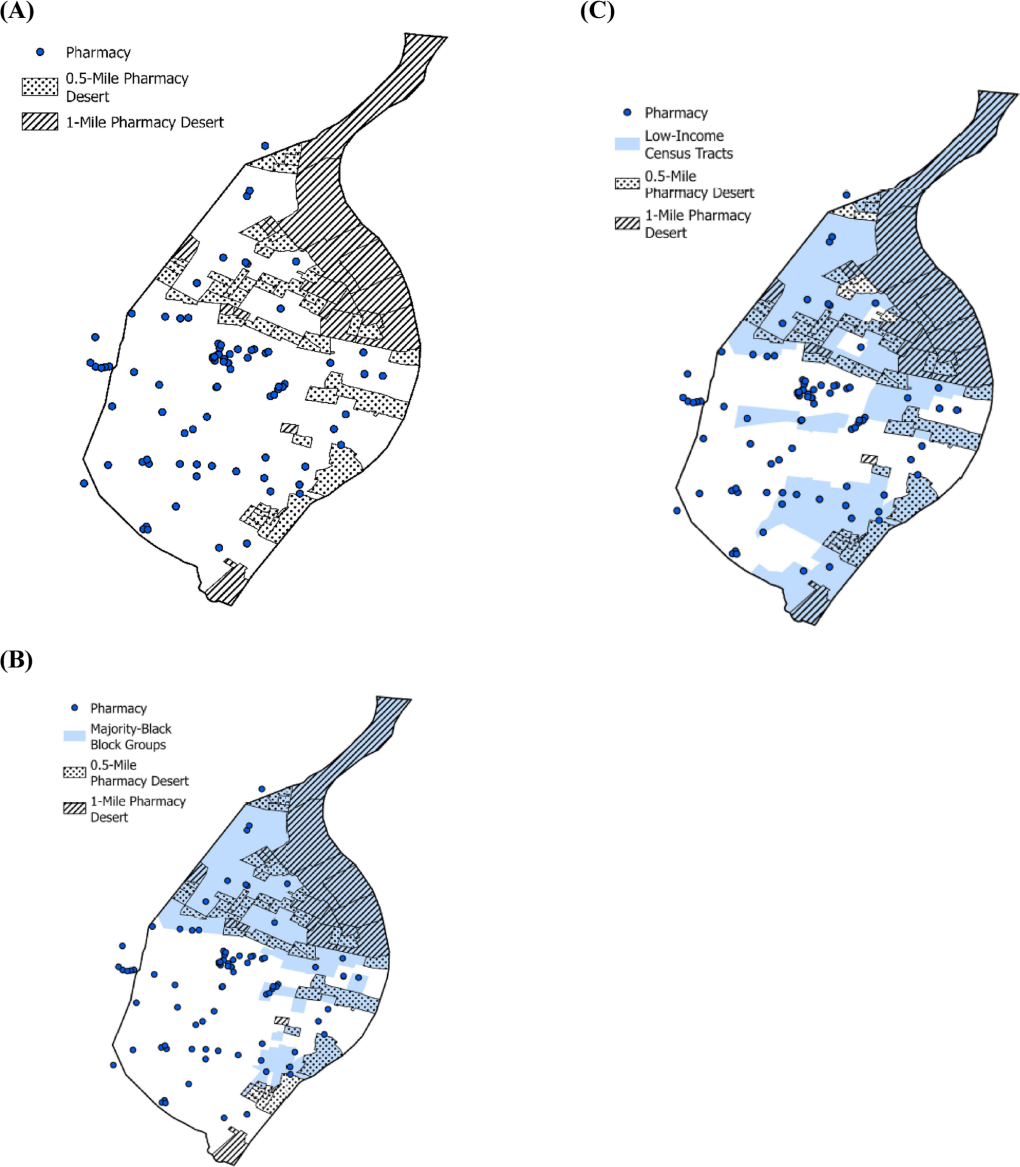
Parts A-C: Pharmacy desert block groups and majority-Black and low-income areas in St. Louis City [20–22, 24–26, 34, 38, 39] **(A)** Pharmacy desert block groups ^a−d^**(B)** Pharmacy desert and majority-Black block groups ^a−d^**(C)** Pharmacy desert block groups and low-income census tracts ^a−d^. ^a^Pharmacy deserts were defined as block-group centroids ≥ 1 mile from the nearest licensed pharmacy or ≥ 0.5 mile where ≥ 20% of households lack a vehicle [24, 34].^b^ Race/ethnicity variables were drawn from the 2020 Decennial Census [25, 26]. “Non-majority-Black” includes block groups that are majority-White or have no single-race majority.^c^ Census-tract income measures were drawn from the 2023 ACS 5-year estimates [20–22].^d^ Low-income tracts were those with ≥ 20% of residents at or below the federal poverty level, or tract median family income ≤ 80% of the St. Louis MSA median; all others were classified as high-income [38, 39]

**Fig. 2:**
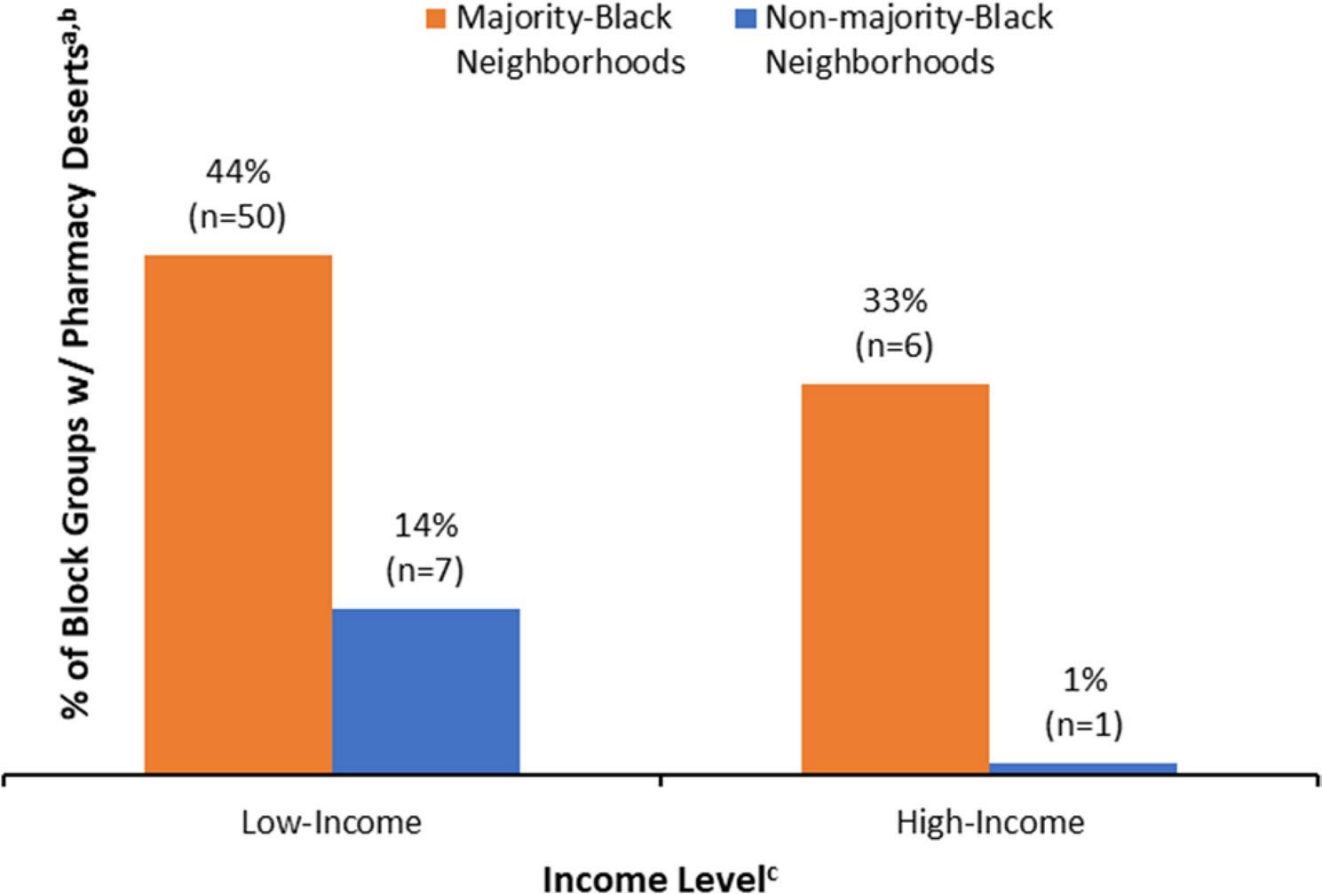
Income as an Effect Modifier of the Relationship Between Race and Pharmacy Desert Prevalence: St. Louis City, 2023 [20–22, 24–26, 34, 38, 39]^a−d^. ^a^ Pharmacy deserts were defined as block-group centroids ≥ 1 mile from the nearest licensed pharmacy or ≥ 0.5 mile where ≥ 20% of households lack a vehicle [24, 34]. ^a^ Pharmacy deserts were defined as block-group centroids ≥ 1 mile from the nearest licensed pharmacy or ≥ 0.5 mile where ≥ 20% of households lack a vehicle [24, 34]. ^b^ Race/ethnicity variables were drawn from the 2020 Decennial Census [25, 26]. “Non-majority-Black” includes block groups that are majority-White or have no single-race majority. ^c^ Census-tract income measures were drawn from the 2023 ACS 5-year estimates [20–22]. ^d^ Low-income tracts were those with ≥ 20% of residents at or below the federal poverty level, or tract median family income ≤ 80% of the St. Louis MSA median; all others were classified as high-income [38, 39]

One in five St. Louis block groups (20.4%; *n* = 64) were classified as pharmacy deserts (Table 2; Fig. 1a–c). Pharmacy deserts were concentrated in majority-Black block groups: 87.5% of deserts were located in these areas, although only 42.4% of all block groups were majority-Black (Fig. 1b). Similarly, 89.1% of deserts were in low-income census tracts, even though just 53.2% of block groups were in low-income areas (Fig. 1c). At the block-group level, 42.1% of majority-Black block groups met the pharmacy-desert definition, compared with 4.4% of non-majority-Black block groups. Among block groups in low-income census tracts, 34.1% met the definition versus 4.8% in high-income tracts. Observed patterns also indicate effect modification by income: within low-income tracts, deserts were present in 43.5% of majority-Black versus 13.5% of non-majority-Black block groups (absolute difference, + 30.0% points); within high-income tracts, the disparity was 33.3% versus 0.8% (+ 32.5 points) (Fig. 2). Thus, the race disparity appears in both income strata but is especially pronounced in relative terms in high-income areas because pharmacy deserts are nearly absent among high-income, non-majority-Black block groups. Conversely, the income gradient differs by race: among non-majority-Black block groups, deserts were much more common in low-income than high-income areas (13.5% vs. 0.8%), whereas among majority-Black block groups, prevalence was high in both income strata (43.5% vs. 33.3%).

**Table 2:** Observed prevalence of pharmacy deserts by block group Racial and income composition in St. Louis City [20–22, 24–26, 34, 38, 39]

Block group type	All block groups – No. (%)	Pharmacy desert block groups- No. (%)^b^	Prevalence of pharmacy desert block groups^a^
Total	314 (100%)	64 (100%)	20.4%
Race^b^			
Majority-Black	133 (42.4%)	56 (87.5%)	42.1%
Non-majority-Black	181 (57.6%)	8 (12.5%)	4.4%
Income^c^			
Low-income	167 (53.2%)	57 (89.1%)	34.1%
High-income	147 (46.8%)	7 (10.9%)	4.8%
Within low-income block groups^c, d^			
Majority-Black^b^	115 (86.5%)	50 (87.7%)	43.5%
Non-majority-Black ^b^	52 (28.7%)	7 (12.3%)	13.5%
Within high-income block groups^c, d^			
Majority-Black^b^	18 (13.5%)	6 (85.7%)	33.3%
Non-majority-Black^b^	129 (71.3%)	1 (14.3%)	0.8%

Note: Prevalence is the percent of block groups in each row classified as pharmacy deserts

^a^ Pharmacy deserts were defined as block-group centroids ≥ 1 mile from the nearest licensed pharmacy or ≥ 0.5 mile where ≥ 20% of households lack a vehicle [24, 34]

^b^ Race/ethnicity variables were drawn from the 2020 Decennial Census [25, 26]. “Non-majority-Black” includes block groups that are majority-White or have no single-race majority

^c^ Census-tract income measures were drawn from the 2023 ACS 5-year estimates [20–22]

^d^ Low-income tracts were those with ≥ 20% of residents at or below the federal poverty level, or tract median family income ≤ 80% of the St. Louis MSA median; all others were classified as high-income [38, 39]

The univariate Poisson regression model revealed that pharmacy deserts were 9.53 times more prevalent in majority-Black block groups (PR = 9.53; 95% CI: 3.17–28.6; *p* < 0.001) and 7.17 times more prevalent in block groups located in low-income census tracts (PR = 7.17; 95% CI: 2.36–21.8; *p* < 0.001) (Table 3). Income significantly modified the race–desert association (cluster-robust Wald F(1, 103) = 4.53, *p* = 0.036; Table 3; Fig. 2). Within low-income census tracts, majority-Black block groups had over three times the prevalence of pharmacy deserts (PR = 3.23; 95% CI, 1.03–10.16; *p* = 0.045), whereas within high-income census tracts the disparity was larger (PR = 43.0; 95% CI, 5.08–364.2; *p* < 0.001) but imprecise given the small number of pharmacy deserts in higher-income areas.

**Table 3:** Prevalence ratio of pharmacy deserts by block group Racial and income composition from Poisson models [20–22, 24–26, 34, 38, 39]

	Pharmacy desert prevalence ratio (95% CI)	*p*-value
Race (overall): Majority-Black vs. non-majority-Black block groups^a−d^	9.53 (3.17–28.6)	< 0.001
Income (overall): Low-income vs. high-income tract block groups^a, c−d^	7.17 (2.36–21.8)	< 0.001
Race (within low-income tracts): Majority-Black vs. non-majority-Black block groups^a−d^	3.23 (1.03–10.2)	0.045
Race (within high-income tracts): Majority-Black vs. non-majority-Black block groups^a−d^	43.0 (5.08–364.2)	< 0.001

Note: Modified Poisson (log link) with robust standard errors clustered by census tract (sandwich). Interaction tested via cluster-robust Wald F-test (race × income): F(1, 103) = 4.53, *p* = 0.036

^a^ Pharmacy deserts were defined as block-group centroids ≥1 mile from the nearest licensed pharmacy or ≥0.5 mile where ≥20% of households lack a vehicle [24, 34]

^b^ Race/ethnicity variables were drawn from the 2020 Decennial Census [25, 26]. “Non-majority-Black” includes block groups that are majority-White or have no single-race majority

^c^ Census-tract income measures were drawn from the 2023 ACS 5-year estimates [20–22]

^d^ Low-income tracts were those with ≥20% of residents at or below the federal poverty level, or tract median family income ≤80% of the St. Louis MSA median; all others were classified as high-income [38, 39]

### Geographic concentration

**Fig. 3:**
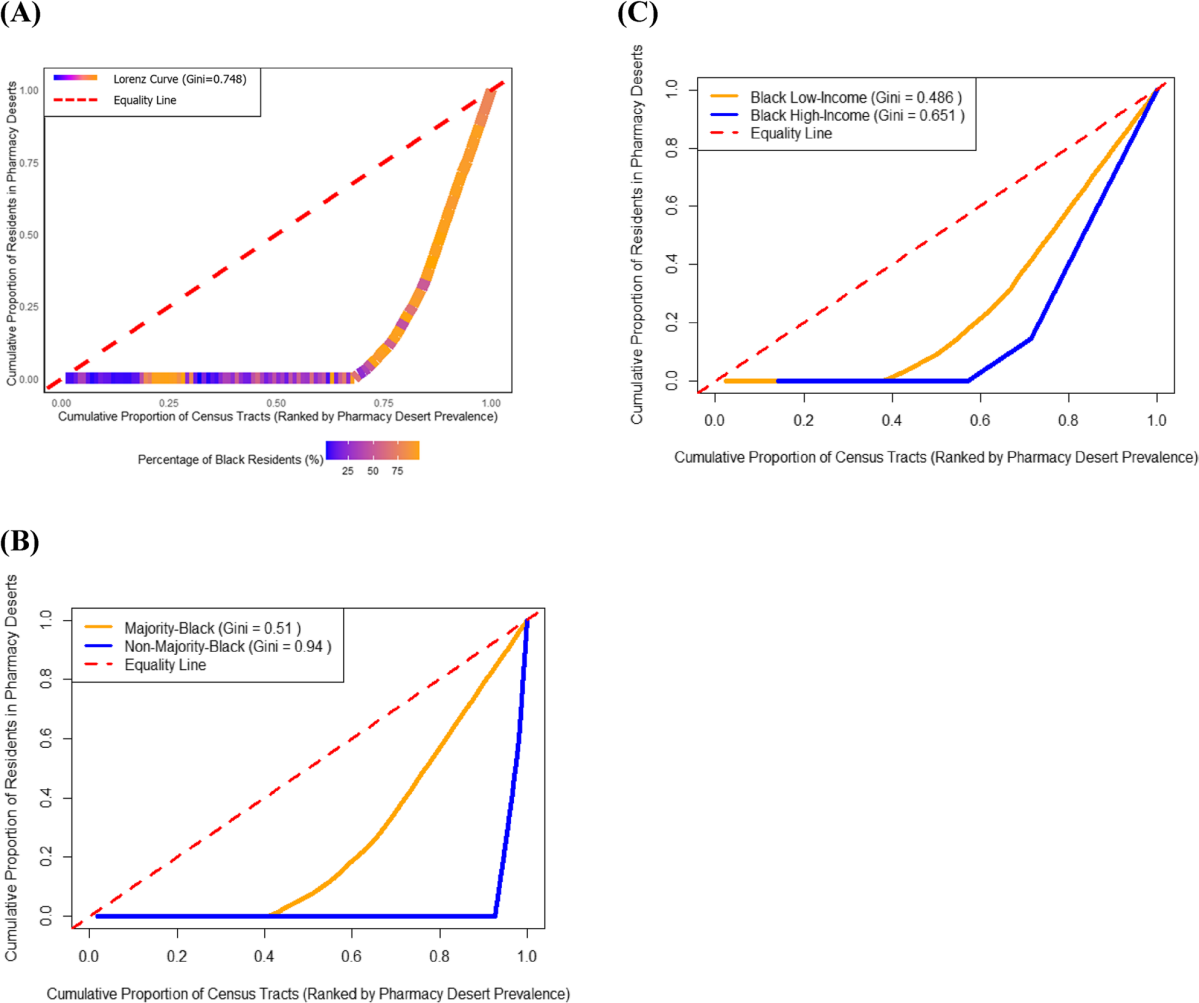
Parts A-C: Lorenz curves showing the distribution of residents living in pharmacy-desert block groups across census tracts: St. Louis City, 2023 [20–22, 24–26, 34, 38, 39]. **(A)** Overall distribution of pharmacy desert residents across census tracts^a−d^. **(B)** Distribution of pharmacy desert residents by majority-Black versus non-majority-Black census tracts^a−d^.**(C)** Distribution of pharmacy desert residents by income level among majority-Black census tracts^a−d^. ^a^ Pharmacy deserts are defined as census tract block groups with centroids one mile or farther from the nearest licensed pharmacy [34] or census tract block groups with centroids a half mile or farther from the nearest licensed pharmacy where 20% of households or more are estimated to not have an available vehicle [24]. ^b^ The demographic and socioeconomic variables for race and ethnicity were drawn from the 2020 Decennial Census [25, 26]. ^c^ Census tract household income level, census tract median family income, and St. Louis MSA median family income was based on the 2023 American Community Survey (ACS) 5-year estimates [20–22]. ^d^ Low-income census tracts were defined as those where 20% or more of the population lived at or below the federal poverty level, or where the tract’s median family income did not exceed 80% of the metropolitan area’s median family income. All other census tracts were classified as high-income [38, 39]

Lorenz curves show the distribution of residents living in pharmacy-desert block groups across census tracts. The Lorenz curve in Fig. 3a shows that pharmacy desert residents are heavily concentrated in a small subset of census tracts, most of which have a high percentage of Black residents. Nearly 70% of census tracts contribute no residents to the cumulative count of pharmacy desert residents, as indicated by the flat portion of the curve. Around 90% of the pharmacy desert population was concentrated within just 28% of census tracts. Among these tracts, 73% had populations that were at least 89% Black. The Gini coefficient of 0.75 also indicates a very high level of inequality.

As shown in Fig. 3b, pharmacy deserts are unequally distributed across both majority-Black and non-majority-Black census tracts, though the patterns differ by racial composition. The high Gini coefficient for non-majority-Black tracts (Gini = 0.94) reflects extreme geographic concentration, with a small number of tracts accounting for most pharmacy desert residents. In contrast, majority-Black tracts exhibit a more dispersed but still inequitable distribution (Gini = 0.51).

Figure 3c further stratifies majority-Black tracts by income level. A steeper Lorenz curve and higher Gini coefficient among higher-income, majority-Black tracts (Gini = 0.65) indicate that these areas, though fewer in number, bear a disproportionate share of pharmacy access barriers. Meanwhile, low-income, majority-Black tracts show more widespread, less concentrated, distribution of pharmacy desert residents (Gini = 0.49).

### Sensitivity to alternative pharmacy access metrics and models

Pharmacies per capita showed no clear patterning by block-group racial composition or census-tract income in univariate tests (all *p* > 0.60). When pharmacies per capita was added to the regression models, first alone and then with additional sociodemographic covariates, estimates of race- and income-related disparities in pharmacy deserts were attenuated and in some cases were no longer statistically significant; however, the overall pattern of higher pharmacy-desert prevalence in majority-Black and low-income areas remained similar. By contrast, higher population density was inversely associated with pharmacy-desert status (PR per 1-SD increase in log density = 0.66; 95% CI: 0.58–0.77; *p* < 0.001). Importantly, after adjusting for density, and again after adding sociodemographic covariates, disparities by race and income persisted, indicating that inequities were not solely attributable to density differences between neighborhoods.

### Sensitivity to pharmacy proximity and vehicle access cutoffs

Results were consistent across alternative fixed distance bands. As the radius increased from 0.5 to 2.0 miles (without the vehicle-access rule), overall desert prevalence declined, yet racial disparities persisted wherever deserts remained. Prevalence ratios for majority-Black versus non–majority-Black block groups were significant at 0.5 miles (PR = 1.73; 95% CI: 1.18–2.53; *p* = 0.005), 0.75 miles (PR = 2.13; 95% CI: 1.28–3.54; *p* = 0.003), and 1.0 mile (PR = 7.48; 95% CI: 1.63–34.4; *p* = 0.010). The income disparity (low vs. high income) was also significant at 1.0 mile (PR = 10.56; 95% CI: 1.35–82.38; *p* = 0.025). At larger radii (≥ 1.25 miles), the reference groups had zero deserts, yielding non-estimable PRs despite clear differences in prevalence. Findings were likewise robust to the vehicle-access cutoff: lowering the threshold from 30% to 10% reduced overall prevalence as expected, but the direction and general pattern of racial and income disparities were similar across cutoffs. At stricter thresholds, point estimates were larger but confidence intervals were wide and sometimes included the null, reflecting fewer block groups meeting the definition.

## Discussion

Our analysis revealed marked racial and socioeconomic disparities in pharmacy access across St. Louis City. Residents of majority-Black and low-income block groups were substantially more likely to live in pharmacy deserts than those in other areas. Racial disparities persisted after accounting for income, with statistically significant differences observed in both low- and high-income areas, although estimates were less precise in stratified analyses. Lorenz curve analyses showed that pharmacy desert residents were concentrated in a small number of census tracts, most of which were majority-Black. These areas were also more likely to experience socioeconomic disadvantage, including higher rates of Medicaid enrollment, lower educational attainment, and limited access to a personal vehicle. Together, these findings underscore the intersection of racial and socioeconomic inequities in limiting access to essential healthcare infrastructure. They build on the 2022 analysis of pharmacy deserts in St. Louis by examining how income, race, and other demographic factors interact [18], and align with national trends [1, 4–12], particularly those reported by Guadamuz et al. (2024), who highlighted the heightened risk of pharmacy closures in Black neighborhoods, even after adjusting for other socioeconomic factors [41].

Sensitivity analyses showed that our findings were generally robust to alternative pharmacy-desert definitions and model specifications. Our primary models were specified a priori to describe inequities in pharmacy-desert status by neighborhood race and income and therefore did not include population density, which may operate as a contextual factor on the pathway between income and access. To assess robustness, we evaluated density and alternative access metrics in secondary models. Substituting an alternative access metric (pharmacies per capita) did not reproduce the strong race- and income-related gradients observed with our distance-based definition, and adding pharmacies per capita to the distance-based models attenuated but did not eliminate racial and income disparities. By contrast, higher population density was inversely associated with pharmacy-desert status (PR = 0.66 per 1-SD increase in log density; 95% CI: 0.58–0.77; *p* < 0.001), and disparities by race and income remained elevated after adjustment for density and, in a further specification, after adding additional sociodemographic covariates, although estimates were less precise in fully adjusted models. Results were also similar when we varied the fixed distance bands (0.5–2.0 miles, without the vehicle-access rule) and when we substituted alternative thresholds for the household vehicle-access component of the composite definition. Overall prevalence changed as expected, but the direction and general pattern of racial and income gaps were similar across cutoffs. Collectively, these checks indicate that the observed inequities are not artifacts of a particular distance band, vehicle-access cutoff, or modeling choice.

The concentration of pharmacy deserts in majority-Black and low-income areas raises critical health equity concerns, particularly as pharmaceuticals play an increasingly vital role in managing health amid growing reliance on medication to treat chronic diseases in an aging population [42, 43]. Despite experiencing higher mortality rates from chronic diseases, Black and Latino individuals are prescribed significantly fewer medications over the course of their lives than their White counterparts, according to national data [44]. Local surveillance data align with these patterns, showing large Black–White gaps across multiple chronic conditions, with emergency-department visit rates among Black residents roughly 2–8 times higher than among White residents (2014–2022) and mortality rates about 1.3–1.9 times higher (2017–2022) [45].

Pharmacy deserts have been shown to significantly hinder medication adherence, particularly among older adults. A national cohort study found that pharmacy closures were associated with sharp declines in adherence to cardiovascular medications among individuals aged 50 and older, with these reductions persisting for at least 12 months [10]. The effects were most pronounced among the oldest adults in the cohort. Additionally, Qato (2017) reported significantly higher rates of underuse of prescription medications due to costs, such as skipping doses or taking less medication than prescribed to stretch a prescription, among residents of predominantly Black and Hispanic low-income communities in Chicago, particularly those living more than a mile from the closest pharmacy [8]. Medication underuse can hinder the management of chronic and infectious diseases and increase rates of emergency room visits and hospitalizations.

The growing integration of primary care services into pharmacies, such as medication management and immunizations, may widen existing disparities in majority-Black and low-income neighborhoods [46]. Pharmacist-led medication management improves health outcomes and reduces costs by optimizing medication types and dosages and preventing drug interactions, particularly for older adults [47]. As pharmacies have increasingly become vaccination sites since 1984 [48], their role has expanded, with 28.2% of U.S. adults receiving flu shots at pharmacies by 2015 [49] and three in five Americans now preferring pharmacy-based vaccinations [50]. Greater pharmacy access has been linked to higher immunization rates [51], while limited evidence suggests pharmacy deserts hinder vaccine uptake. For example, breast cancer patients in pharmacy deserts had lower flu vaccination rates despite insurance coverage and elevated risk for influenza complications [49]. In St. Louis, early COVID-19 vaccination rates were lower among Black and Hispanic residents in high–Social Vulnerability Index areas but improved once pharmacies and community-based sites became key distribution points, illustrating the potential for pharmacy-based interventions to reduce disparities [52].

The City of St. Louis Department of Health plans to use these findings to guide educational outreach initiatives in pharmacy desert areas, aiming to raise awareness among residents about medication delivery services, telehealth options, and other healthcare resources available at nearby pharmacies. These efforts will be conducted in coordination with a local community pharmacy, with the first phase prioritizing senior citizen and assisted living centers. Evidence underscores the value of interventions such as promoting mail-order pharmacy services to enhance medication adherence, particularly in pharmacy deserts. Studies have found that adherence declines are smaller among individuals who use mail-order services after local pharmacies close [10]. Linking Medicaid patients to eligible transportation services and educating residents about pending pharmacy closures and the safety of mail-order services could further improve access [10, 53].

While this study provides important insights, it has several limitations. Using block group centroids instead of individual residential addresses to measure proximity may have introduced some misclassification of pharmacy deserts; however, this bias is likely random rather than systematic. The analysis did not incorporate network-based travel distances, which may underestimate the actual distance to the nearest pharmacy, nor did it account for alternative access methods such as home delivery. It also assumed that all residents would use the nearest pharmacy, without considering individual preferences for in-clinic, hospital-based, or chain pharmacies, some of which may fall outside the defined buffer. Specialty pharmacies were included despite serving more limited populations, and many pharmacies located near pharmacy desert areas lack key services such as extended hours, which can limit their practical usability. These assumptions may lead to an underestimation of pharmacy desert prevalence by not fully capturing which pharmacies are accessible or acceptable to the populations they are intended to serve. In addition, some prevalence ratio estimates were imprecise due to the small number of block groups in these categories, particularly those for high-income, majority-Black block groups. Nevertheless, the magnitude and direction of these associations were consistent with broader trends, suggesting that the observed disparities are not due solely to statistical instability. The study also used two different data sources, the 2020 Decennial Census and the 2023 ACS 5-year estimates, which may not perfectly align. However, any resulting error is unlikely to account for the observed disparities. Despite these limitations, the racial and income disparities in pharmacy access were robust, unlikely to be explained by methodological factors alone, and indicative of the ongoing impact of structural barriers on pharmacy access. Our analysis demonstrates that in St. Louis City, pharmacy deserts are disproportionately concentrated in majority-Black and low-income communities, with Lorenz curves showing that a large share of residents in pharmacy-desert block groups are clustered within a relatively small number of areas of the city. Looking ahead, the City of St. Louis Department of Health will evaluate the impacts of our targeted educational outreach in the most affected areas and our partnership with a local community pharmacy. We also recommend that health departments in other urban and rural jurisdictions incorporate local assessments of pharmacy deserts when evaluating and addressing determinants of health. In addition, subsequent research in St. Louis and elsewhere should assess how living in or near a pharmacy desert affects day-to-day health management and out-of-pocket costs by collecting resident-level measures such as pharmacy travel distance, difficulty obtaining medications due to long wait or processing times related to pharmacy consolidation and closures, frequency of missed or delayed prescription fills, medication adherence, costs related to pharmacy travel, and out-of-pocket price differences for medications. At the policy level, additional solutions should be explored, including enhanced Medicaid reimbursement for prescriptions filled at pharmacies serving underserved areas and closer assessment and regulation of pharmacy benefit manager (PBM) preferred-network practices [41, 54]. Independent community pharmacies often serve neighborhoods adjacent to pharmacy deserts and urban areas with higher concentrations of racial and ethnic minority residents and Medicaid beneficiaries, and they are frequently required to absorb additional demand when nearby chain or retail pharmacies close. Because PBMs set reimbursement terms for health plans, highly concentrated PBM markets can steer patients toward affiliated or “preferred” chain pharmacies and exclude independent community pharmacies, raising out-of-pocket costs at these pharmacies or forcing longer travel for in-network fills. Low reimbursement levels can also make dispensing financially unsustainable for community pharmacies, increasing closure risk and limiting the time pharmacists can spend with patients. Evaluating and addressing how PBM network design and reimbursement policies affect access, costs at the counter, and pharmacy viability should therefore be a central component of any state-level strategy to narrow pharmacy access gaps [41, 54].

## Data Availability

All data used in this study are publicly available. Block-group demographics and census geography shapefiles came from the U.S. Census Bureau (2020 Decennial Census DHC, 2023 ACS 5-year estimates, TIGER/Line Shapefiles). Pharmacy locations were obtained from the Missouri Spatial Data Information Service and updated to reflect closures as of August 2024.
